# Nanoparticle Tracing during Laser Powder Bed Fusion of Oxide Dispersion Strengthened Steels

**DOI:** 10.3390/ma14133463

**Published:** 2021-06-22

**Authors:** Yangyiwei Yang, Carlos Doñate-Buendía, Timileyin David Oyedeji, Bilal Gökce, Bai-Xiang Xu

**Affiliations:** 1 Mechanics of Functional Materials Division, Institute of Materials Science, The Technical University of Darmstadt, D-64287 Darmstadt, Germany; yangyiwei.yang@mfm.tu-darmstadt.de (Y.Y.); timileyin.oyedeji@tu-darmstadt.de (T.D.O.); 2 Materials Science and Additive Manufacturing, School of Mechanical Engineering and Safety Engineering, University of Wuppertal, D-42119 Wuppertal, Germany; carlos.donate-buendia@uni-wuppertal.de (C.D.-B.); goekce@uni-wuppertal.de (B.G.)

**Keywords:** additive manufacturing, laser powder bed fusion, selective laser melting, oxide dispersion strengthened steel, phase-field model, finite element simulation, nanoparticle interaction

## Abstract

The control of nanoparticle agglomeration during the fabrication of oxide dispersion strengthened steels is a key factor in maximizing their mechanical and high temperature reinforcement properties. However, the characterization of the nanoparticle evolution during processing represents a challenge due to the lack of experimental methodologies that allow in situ evaluation during laser powder bed fusion (LPBF) of nanoparticle-additivated steel powders. To address this problem, a simulation scheme is proposed to trace the drift and the interactions of the nanoparticles in the melt pool by joint heat-melt-microstructure–coupled phase-field simulation with nanoparticle kinematics. Van der Waals attraction and electrostatic repulsion with screened-Coulomb potential are explicitly employed to model the interactions with assumptions made based on reported experimental evidence. Numerical simulations have been conducted for LPBF of oxide nanoparticle-additivated PM2000 powder considering various factors, including the nanoparticle composition and size distribution. The obtained results provide a statistical and graphical demonstration of the temporal and spatial variations of the traced nanoparticles, showing ∼55% of the nanoparticles within the generated grains, and a smaller fraction of ∼30% in the pores, ∼13% on the surface, and ∼2% on the grain boundaries. To prove the methodology and compare it with experimental observations, the simulations are performed for LPBF of a 0.005 wt % yttrium oxide nanoparticle-additivated PM2000 powder and the final degree of nanoparticle agglomeration and distribution are analyzed with respect to a series of geometric and material parameters.

## 1. Introduction

Powder-based laser additive manufacturing techniques such as Laser Powder Bed Fusion (LPBF) [1] or Direct Energy Deposition (DED) [2] have been recently established as methods that allow the strengthening of metal alloys by modification of the microstructure [3]. Often, the strengthening is achieved by introducing lattice-matched nanoparticles within the surrounding matrix [1]. Another methodology used for strengthening is the introduction of exogenic dispersoids into the metallic matrix leading to the retardation of dislocation movements. Dislocations interact with the impenetrable dispersoids by the formation of a dislocation loop in between neighboring dispersoids only allowing dislocation loops with equilibrium diameter below the dispersoids interspacing to bypass the obstacle. This effect, known as the Orowan mechanism, describes that a fine dispersion results in efficient hardening [4]. Oxide dispersion strengthened (ODS) steels make use of this mechanism. The improved mechanical properties at high temperatures of ODS alloys can be related to the nature of the steel matrix as well as the composition, size, and distribution of the dispersoids (i.e., nanoparticles). Introduced nanoparticles in ODS steels are typically composed of yttrium-based oxides, exhibiting low solubility in the steel matrix and having a low potential for coarsening by Ostwald ripening [5]. In combination with the refining agent Titanium, nanoparticles with the chemical composition $Y_{2}Ti_{2}O_{7}$ [6], $Y_{2}TiO_{5}$ [6] are formed [7] with typical diameters of 2–3 nm in the 14YWT alloy [8]. The addition of aluminum enables the formation of various Y-Al-O-based compounds ($YAlO_{3}$, $Y_{3}Al_{5}O_{1}2$) [9] leading to coarser dispersoids (∼15 nm) and a reduced number density in the PM2000 alloy [8], resulting in increased ductility at the cost of lower strength.

The main fabrication route for ODS steels is the powder metallurgy route [10]. However, this expensive and complex fabrication route often leads to a low fracture toughness in the fabricated part and is inflexible in the part design [11]. As an alternative, additive manufacturing techniques are capable of producing ODS materials offering high solidification rates in combination with strong Marangoni forces within the melt pool, potentially leading to a homogeneous distribution of the introduced nanoparticles [12]. The distribution of these nanoparticles is highly influenced by the additive manufacturing technique employed and the parameters selected for processing due to differences in the melt pool dynamics. However, recent studies on this topic show that it is difficult to achieve the optimum nanoparticle size according to the Orowan mechanism due to segregation or agglomeration of the nanoparticles, which in turn was found to deteriorate the mechanical properties of the part [13]. Approaches to optimize the process such as evaluating the influence of the powder characteristics [14] and process parameters [15], or alternative additivation routes such as light mixing [16], improve the dispersion of the nanoparticles; however, they still lack control over the nanoparticle size.

The size and dispersion of the nanoparticles in the ODS steels are influenced during the steps that the initial powder undergoes until the fabrication of the ODS steel. First, the nanoparticles are supported on the steel microparticles. Ball milling of the yttrium-based nanoparticles with the steel powder is the most common approach to achieve it [17,18]. While widely employed, the control of the final nanoparticle size and degree of dispersion by this methodology is limited, and the steel microparticles size and shape can also be affected [19]. To address this drawback, alternative supporting procedures have been proposed [20] such as resonant acoustic mixing [21], solid–liquid reaction [22], or colloidal dielectrophoretic deposition [23,24]. Once the nanoparticle-additivated powder is obtained, the processing technique employed to generate the ODS steel samples and the experimental parameters selected determine the evolution of the nanoparticles, their final size, and dispersion in the ODS steel [12,25]. Even techniques with a similar working principle to LPBF like direct energy deposition (DED)—both laser additive manufacturing techniques—lead to differences in the nanoparticle agglomeration during processing due to the higher cooling rates of the melt pool achieved in LPBF that favor the preservation of the nanoparticle dispersion [12]. Even though these experimental observations provide an insight into the nanoparticle behaviors, further investigations would be required to completely understand the undergoing nanoparticle capture (nanoparticles trapped interior the microstructure), enrichment (local concentration increase of nanoparticles), and agglomeration processes [26,27,28,29]. Since the nanoparticles are not accessible during processing to perform in situ measurements, the combination of simulations with the experimental characterization of the nanoparticle-additivated powder and the generated ODS steel [13,30] represents the best approach to understand and control the LPBF processing of ODS steels.

Intuitively, the simulation of the LPBF processing of ODS steels and the underlying behaviors of nanoparticles, including their drift in the melt pool, captured during re-solidification, enrichment, and agglomeration, requires the proper modeling of the physical phenomena during the LPBF process and interactions among nanoparticles. It already remains a great challenge to model the underlying phenomena due to their sophisticated and interactive nature, covering a broad range of time and length scales. Notably, the thermal/mass transfers and material transformations (including melting, solidification and evaporation) dominate on a length scale of hundreds to thousands micrometers over a few dozen milliseconds. However, nanoparticle attraction and repulsion take place in a different spatial and temporal scale, around several tenths of nanometers and within microseconds. Finally, the manufacturing of an ODS steel sample is explained in a completely different scale of centimeters and takes hours or even days [31]. Resultant morphologies also reveal themselves in a multiscale fashion [32]. In this sense, the existent simulation schemes for the LPBF process are more or less subjected to strong simplifications and segregated modeling schemes, considering either only selected aspects or with the thermal history taken as input from separate numerical approaches. Those schemes usually feature a computational fluid dynamics method to simulate the thermal-fluid coupled spatial-transient evolution, including the arbitrary Lagrangian-Eulerian [33,34,35,36] and Lattice Boltzmann [37,38,39], incorporated with another method to simulate the accompanied microstructure evolution (mostly the polycrystalline re-solidification), such as the cellular automata [40,41,42,43,44] and phase-field [45]. Recently, we proposed a new phase-field model considering coupled processes among heat transfer, melt flow dynamics, and microstructure evolution (noted as “heat-melt-microstructure–coupled processes"), which shows the possibility to simulate the LPBF process in a unified and thermodynamic consistent route and the ability to recapitulate various experimental observations via simulation, such as high-gradient temperature field, tilted columnar grains, and lack-of-fusion pores with irregular shape [46].

On the other hand, very few works have been conducted regarding nanoparticle drift and interactions in the melt pool, which is, however, the central aspect to investigate the behaviors such as enrichment and agglomeration. Xu et al. [29,47] aimed to convey fundamental understandings regarding nanoparticle capture during re-solidification in order to obtain a nanocomposite-dispersed metal bulk, bringing the consideration of the Van der Waals (abbreviated as VDW hereinafter) effect, the Brownian effect, and thermodynamic analysis into the modeling of interactions in the nanocomposite-melt dispersion system. There are also experimental works revealing the VDW [48] and electrostatic interactions [49,50,51] among inorganic nanocomposites in the liquid metal. Nevertheless, those works disregard the influence of the driving effect from the melt flow dynamics when describing the nanocomposite-liquid/molten metal dispersion as well as the inter-particle interactions. In this sense, a simulation scheme combining the coupled phenomena (especially the melt pool dynamic and microstructure evolution) and the nanoparticle behaviors is still to be developed for the investigation of LPBF processing of ODS steels.

Joining our heat-melt-microstructure (HMM) coupled phase-field model with nanoparticle kinematics, we present and apply in this work a simulation scheme for tracing the nanoparticle drift and interactions in the melt pool during the LPBF process of the ODS steels. This proposed scheme is aimed to demonstrate the chronological and spatial variation of multiple traced nanoparticles with respect to various factors (such as chemical composition and size distribution), which enables graphical and statistical analysis on the nanoparticle migration and further effects, such as nanoparticle capture, enrichment, and agglomeration. The nanoparticle compositions used in the simulation are chosen based on our experimental studies on the additive manufacturing of ODS steels, including the use of $Y_{2}O_{3}$ [12,23] and YIG (yttrium iron garnet, $Y_{3}Fe_{5}O_{1}2$) [23] nanoparticles. Influences from different types of nanoparticle size distribution, including the monomodal, normal, and log-normal distributions fitted from the experimental measurement [23], are also investigated and discussed in this work.

## 2. Models

### 2.1. Non-Isothermal Phase-Field Model for Stable LPBF Processes

**Figure 1:**
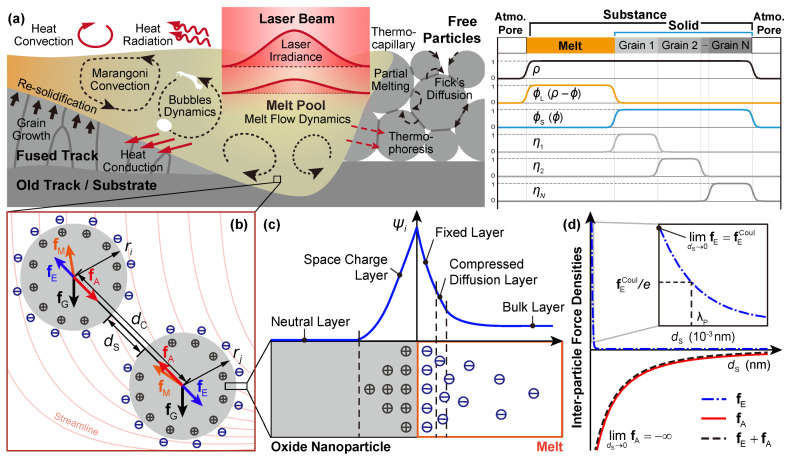
(**a**) Schematic of interactive physical phenomena during a stable LPBF process (left) and the order parameter profiles of the phase-field model (right); (**b**) force analysis of two oxide nanoparticles (labeled as *i* and *j*) in the melt pool with streamline and charges denoted; (**c**) schematic of charge distribution and potential profile across the oxide-melt interface based on explanations in [49]; (**d**) variation of the inter-particle force densities to the surface distance.

To properly simulate the microstructure of a polycrystalline material, manufactured under a stable LPBF process, a conserved order parameter (OP) ρ is employed to represent the substance and atmosphere/pores; a set of non-conserved OPs $\phi_{S}$ and $\phi_{L}$ is employed to represent the solid and liquid phases, respectively; and a series of non-conserved OPs $\left\{\eta_{\alpha }\right\}$ are employed to represent the orientation distribution among the powders/grains. Two constraints should be applied on those OPs to properly recapture the reality (Figure 1a), i.e., substance constraint $\left(1-\rho \right)+\phi_{L}+\phi_{S}=1$ to restrict the existence of liquid as well as solid within the substance only, and the polycrystal constraint $\left(1-\phi_{S}\right)+\sum_{\alpha }\eta_{\alpha }=1$ to restrict polycrystalline orientations inside the solid substance. Notice that the substance constraint always satisfies when $\phi_{L}=\rho -\phi_{S}$. Therefore, only one ϕ to represent the solid substance and (ρ−ϕ) to represent the liquid substance is sufficient. The other constraint can be fulfilled via some practical numerical method, e.g., the Lagrange multiplier or the penalty method.

We consider a stable LPBF process (Figure 1a), i.e., without significant vaporization and resultant phenomena, such as scattering and keyholing. In this sense, the heat/mass transfer through various diffusion paths (e.g., surface, grain interior, and grain boundary) and melt flow, the dynamics of melt flow as well as the bubbles, the melting-solidification of the grains, and some inter-coupling effects, such as thermocapillary (Marangoni effect) and thermophoresis (Soret effect), would take the major role determining the resultant microstructure of the manufactured sample. The nonlinear kinetic system in simulating the stable LPBF processes is then adapted from our HMM-coupled phase-field model [46]. It is worth noting that the HMM-coupled phase-field model, derived under the thermodynamic-consistent framework, can be regarded as the combination of Navier-Stokes-Cahn-Hilliard (NSCH) and Navier-Stokes-Allen-Cahn (NSAC) systems with inter-coupling effects integrated. In this work, however, mentioned inter-coupling effects are tentatively dropped under the consideration of the computational stability, consumption, and complicity control in variants. These effects would be explicitly covered in our separate (e.g., the thermophoresis in [52]) or upcoming works. The adapted nonlinear kinetic system for the velocity field of the melt u, the temperature field *T*, and fields of OPs ρ,ϕ and $\left\{\eta_{\alpha }\right\}$, eventually presents as

(1) ∇·u=0,DuDt=−∇p+1Re∇2u+1Fr2g^,DTDt+1SteϕDϕDt=∇·1PeT∇T+qv,DρDt=∇·1Peρ∇δFδρ,DϕDt=−1AcϕδFδϕ,DηαDt=−1AcηδFδηα,

with dimensionless quantities, namely the Reynolds number (Re), the Froude number (Fr), the Stefan number (Ste), the Péclet numbers for thermal (${\mathrm{Pe}}_{T}$) and mass (${\mathrm{Pe}}_{\rho }$) transfer, and Allen-Cahn numbers for melting-solidification (${\mathrm{Ac}}_{\phi }$) and grain growth (${\mathrm{Ac}}_{\eta }$). Here, $\hat{g}$ stands for the unit vector of gravitation direction. These quantities are employed not only to parameterize the nonlinear system but also to characterize the ratio between their corresponding physical processes to the chosen rate (by default, the characteristic rate of the fluid). Detailed parameterization of these quantities will be explained in Section 3.3.

The explicit formulation of non-isothermal free energy F is formulated as

(2) $$\begin{array}{cc} \; & F=\int_{\Omega }\left[f_{\mathrm{ht}}\left(T,\rho ,\phi ,\left\{\eta_{\alpha }\right\}\right)+f_{\mathrm{loc}}\left(T,\rho ,\phi ,\left\{\eta_{\alpha }\right\}\right)+f_{\mathrm{grad}}\left(\nabla \rho ,\nabla \phi ,\left\{\nabla \eta_{\alpha }\right\}\right)\right]d\Omega .\\ \; & \begin{array}{cc} f_{\mathrm{ht}}\left(T,\rho ,\phi ,\left\{\eta_{\alpha }\right\}\right)= & \xi \left(\underline{A}\rho +\underline{G}\phi +\underline{B}\sum_{\alpha }\eta_{\alpha }\right)\left\{c_{r}\left[T\ln\frac{T}{T_{M}}-\left(T-T_{M}\right)\right]-\right.\\ \; & \left.\Phi_{L}\left[\frac{T-T_{M}}{T_{M}}L\right]\right\}, \end{array}\\ \; & f_{\mathrm{loc}}\left(T,\rho ,\phi ,\left\{\eta_{\alpha }\right\}\right)=w_{\mathrm{ss}}\left(T,\rho \right)+w_{\mathrm{sl}}\left(T,\rho ,\phi \right)+w_{\mathrm{gr}}\left(T,\phi ,\left\{\eta_{\alpha }\right\}\right),\\ \; & \begin{array}{cc} f_{\mathrm{grad}}\left(T,\nabla \rho ,\phi ,\left\{\nabla \eta_{\alpha }\right\}\right)= & \frac{1}{2}T\kappa_{\rho }{\left|\nabla \rho \right|}^{2}+\frac{1}{2}T\kappa_{\phi }{\left|\nabla \phi \right|}^{2}\\ \; & +\frac{1}{2}T\kappa_{\phi }{\left|\nabla \rho -\nabla \phi \right|}^{2}+\frac{1}{2}T\kappa_{\eta }\sum_{\alpha }{\left|\nabla \eta_{\alpha }\right|}^{2}, \end{array} \end{array}$$

with

$$\begin{array}{cc} \; & w_{\mathrm{ss}}\left(T,\rho \right)=\underline{C}\left(T\right)\left[\rho^{2}{\left(1-\rho \right)}^{2}\right],\\ \; & \begin{array}{cc} w_{\mathrm{sl}}\left(T,\rho ,\phi \right)= & \underline{H}\left(T\right)\left[\rho^{2}+6\left(1-\rho \right)\left\{\phi^{2}+{\left(\rho -\phi \right)}^{2}\right\}-4\left(2-\rho \right)\left\{\phi^{3}+{\left(\rho -\phi \right)}^{3}\right\}\right.\\ \; & \left.+3{\left\{\phi^{2}+{\left(\rho -\phi \right)}^{2}\right\}}^{2}\right], \end{array}\\ \; & w_{\mathrm{gr}}\left(T,\phi ,\left\{\eta_{\alpha }\right\}\right)=\underline{D}\left(T\right)\left[\phi^{2}+6\left(1-\phi \right)\sum_{\alpha }\eta_{\alpha }^{2}-4\left(2-\phi \right)\sum_{\alpha }\eta_{\alpha }^{3}+3{\left(\sum_{\alpha }\eta_{\alpha }^{2}\right)}^{2}\right], \end{array}$$

and

$$\begin{array}{cc} \underline{C}\left(T\right) & ={\underline{C}}_{\mathrm{pt}}-{\underline{C}}_{\mathrm{cf}}\left(T-T_{M}\right),\\ \underline{D}\left(T\right) & ={\underline{D}}_{\mathrm{pt}}-{\underline{D}}_{\mathrm{cf}}\left(T-T_{M}\right),\\ \underline{H}\left(T\right) & ={\underline{H}}_{\mathrm{pt}}-{\underline{H}}_{\mathrm{cf}}\left(T-T_{M}\right) \end{array},$$

where $T_{M}$ is the melting temperature, L is the latent heat of the material, and $c_{r}$ is the relative volumetric specific heat. Therefore, the variational derivatives of the free energy appearing in Equation (2) yield

$$\begin{array}{cc} \; & \frac{\delta F}{\delta \rho }=\frac{\partial f_{\mathrm{ht}}}{\partial \rho }+\frac{\partial f_{\mathrm{loc}}}{\partial \rho }-T\kappa_{\rho }\nabla^{2}\rho -T\kappa_{\phi }\left(\nabla^{2}\rho -\nabla^{2}\phi \right),\\ \; & \frac{\delta F}{\delta \phi }=\frac{\partial f_{\mathrm{ht}}}{\partial \phi }+\frac{\partial f_{\mathrm{loc}}}{\partial \phi }-T\kappa_{\phi }\nabla^{2}\phi +T\kappa_{\phi }\left(\nabla^{2}\rho -\nabla^{2}\phi \right),\\ \; & \frac{\delta F}{\delta \eta_{\alpha }}=\frac{\partial f_{\mathrm{ht}}}{\partial \eta_{\alpha }}+\frac{\partial f_{\mathrm{loc}}}{\partial \eta_{\alpha }}-T\kappa_{\eta }\nabla^{2}\eta_{\alpha }. \end{array}$$

Model parameters $\underline{A},\underline{B},\underline{G},{\underline{C}}_{\mathrm{pt}},{\underline{C}}_{\mathrm{cf}},{\underline{D}}_{\mathrm{pt}},{\underline{D}}_{\mathrm{cf}},{\underline{H}}_{\mathrm{pt}},{\underline{H}}_{\mathrm{cf}}$, as well as the gradient constants ($\kappa_{\rho }$, $\kappa_{\phi }$, and $\kappa_{\eta }$), are obtained from given diffusive interface width and the experimentally measured interface energies. Coefficient ξ is employed to favor the determination of model parameters by fitting the experimental results (sufficiently summarized in Ref. [46] and supplementary information of Ref. [53]). $\Phi_{L}$ is the interpolation function indicating the spatial landscape of the liquid/melt (see Section 3.3).

Finally, the thermal effect is equivalently treated as an internal heat source term $q_{v}$ moving with the scan velocity $v_{l}$

(3) $$\begin{array}{cc} q_{v} & =\Phi_{\mathrm{ss}}\beta P_{l}\left\{\frac{\Pi }{\pi R_{l}^{2}}\exp\left[-\Pi \frac{|x-v_{l}{t|}^{2}}{R_{l}^{2}}\right]\right\}\frac{2}{\zeta \sqrt{\pi }}\left(-\frac{|z-z_{v}{|}^{2}}{\zeta^{2}}\right), \end{array}$$

in which $P_{l}$ is the laser beam power reaching the surface of the powder bed, β is the attenuation coefficient. $\Phi_{\mathrm{ss}}$ is the interpolation function for the substance. x is an arbitrary point on the projected plane of the laser beam on the powder bed surface, while *z* is an arbitrary depth from the plane. $\left(v_{l}t,z_{v}\right)$ is the moving center of the beam following the morphology of the surface. ζ is the characteristic penetration depth and normally takes the value of the powder bed thickness. Notice that parameter Π is utilized to adjust the concentration of the deposited power inside the circular beam spot with nominal radius $R_{l}$, e.g., as suggested by the ISO standard [54], Π=2, indicating 86.5% of the concentrated power within the spot. $P_{l}$, $R_{l}$, and the mode of the scan velocity $v_{l}=\left|v_{l}\right|$ (scan speed) are thereby regarded as the major processing parameters of the laser scan in this work.

### 2.2. Nanoparticle Kinematics

The coupled evolution among polycrystalline microstructure, melt flow dynamics, and temperature transfer is calculated on the mesoscale (0.1–100 μm) using the phase-field model presented above. Since the size of nanoparticles is smaller by several orders, the possible influence of nanoparticles on these mesoscale effects is ignored. As an important part of nanoparticle kinematics, the drift effect of melt flow is inherited from the phase-field simulations. Moreover, nanoparticles can interact with each other by different mechanisms. Based on former research for dispersed non-metallic particles [29,47,55,56,57] neglecting the rotation, the kinematic equation for a dispersed rigid nanoparticle (labeled as *i*) with a volume $V_{i}$, the density $\varrho_{i}$, and the translation velocity $v_{i}$ in the melt,

(4) $$\varrho_{i}\frac{\;dv_{i}}{\;dt}=\varrho_{i}g+f_{M}+f_{A}+f_{E},$$

in which the r.h.s. terms are the driving force densities due to the gravity, the melt flow, the VDW, and the electrostatic interaction, respectively (Figure 1b).

Considering the boundary of the nanoparticle as $\partial V_{i}$, the force density due to melt-flow driven is formulated as $f_{M}=\frac{1}{V_{i}}\int_{\partial V_{i}}\sigma \;\cdot \;ndS$, in which the Cauchy stress tensor of the fluid reads σ=−pI+1Re∇2u. This is widely employed in simulating nanoparticle dispersion, including lattice-Boltzmann [57,58] and finite element method using static [59] or fictitious domain [60,61]. Assuming an infinitesimal size of the nanoparticle compared to the characteristic length scale of the melt, i.e.,  $V_{i}\to 0$, $f_{M}$ can be thus calculated as follows in this work according to the definition of the divergence

(5) $$\begin{array}{cc} f_{M} & =\lim_{V_{i}\to 0}\frac{1}{V_{i}}\int_{\partial V_{i}}{\sigma \;\cdot \;n\;dS=\nabla \;\cdot \;\sigma |}_{x_{i}}\\ \; & =\varrho_{m}\left({\left.\frac{\;Du}{\;Dt}\right|}_{x_{i}}-\frac{1}{{\mathrm{Fr}}^{2}}\hat{g}\right), \end{array}$$

where $x_{i}$ is the center point of the nanoparticle, and ${\left.\;Du/\;Dt\right|}_{x_{i}}$ is the undisturbed time difference of the melt velocity at point $x_{i}$.

The VDW and electrostatic force densities, depending on respective inter-particle potential $U_{A}$ and $U_{E}$, can be formulated as

(6) $$f_{A}=-\frac{1}{V_{i}}\nabla_{d}U_{A}\left(d_{C}\right),\;f_{E}=-\frac{1}{V_{i}}\nabla_{d}U_{E}\left(d_{C}\right),$$

where $d_{C}$ is the inter-particle center distance, $d_{C}=\left|x_{i}-x_{j}\right|$, distinguishing for the surface distance $d_{S}=\left|x_{i}-x_{j}\right|-\left(r_{i}+r_{j}\right)$, as shown in Figure 1b. $\nabla_{d}$ represents the gradient operator in the inter-particle direction. Derived by Hamaker [62], the inter-particle potential of VDW attraction between nanoparticles *i* and *j* with corresponding radius $r_{i}$ and $r_{j}$ is

(7) $$U_{A}\left(d_{C}\right)=-\frac{A_{p}:m}{6}\left\{\frac{2r_{i}r_{j}}{d_{C}^{2}-{\left(r_{i}+r_{j}\right)}^{2}}+\frac{2r_{i}r_{j}}{d_{C}^{2}-{\left(r_{i}-r_{j}\right)}^{2}}+\ln\left[\frac{d_{C}^{2}-{\left(r_{i}+r_{j}\right)}^{2}}{d_{C}^{2}-{\left(r_{i}-r_{j}\right)}^{2}}\right]\right\},$$

where $A_{p}:m$ is referred to as the Hamaker coefficient of the nanoparticle in a medium, which is dependent on the permittivity of involved materials and the intervening medium. According to Dzyaloshinskii-Lifshitz-Pitaevskii interpretation [63,64], in which nanoparticles are treated as continuous media (rather than atomic structure as [62]), and inter-particle forces are derived in terms of permittivities and refractive indices, the non-retarded (instantaneous) Hamaker coefficient for VDW interactions between two nanoparticles of the same material through a medium (denoted as $A_{p}:m$) is estimated as

(8a) $$A_{p}:m=\frac{3}{4}k_{B}T{\left(\frac{\varepsilon_{p}-\varepsilon_{m}}{\varepsilon_{p}+\varepsilon_{m}}\right)}^{2}+\frac{3\hbar \omega_{e}}{16\sqrt{2}}\frac{{\left(n_{p}^{2}-n_{m}^{2}\right)}^{2}}{{\left(n_{p}^{2}+n_{m}^{2}\right)}^{3/2}}$$

with the permittivities $\varepsilon_{p}$ and $\varepsilon_{m}$, and the refractive indices $n_{p}$ and $n_{m}$ of the nanoparticle and the medium, respectively. $k_{B}$ and *ℏ* are Boltzmann constant and reduced Planck constant, while $\omega_{e}$ is the electronic absorption frequency, ranging in $3∼5\times 10^{15}\;\mathrm{Hz}$. It is worth noting that Equation (8a) is not employable due to the difficulties in the practical measurement of permittivity and refractive index of the molten metal. As an alternative, $A_{p}:m$ is calculated from the ones for nanoparticle ($A_{m}:v$) and medium ($A_{p}:v$) that obtained separately in the vacuum, according to the following combining relation [48,65]

(8b) $$A_{p}:m={\left(\sqrt{A_{p}:v}-\sqrt{A_{m}:v}\right)}^{2},$$

where $A_{p}:v$ can be obtained from experimentally measured optical data [56], while $A_{m}:v$ for dispersed metallic particle/droplet in the vacuum is derived from Dzyaloshinskii-Lifshitz-Pitaevskii theory by taking the estimated frequency-dependent permittivity for metals as $\varepsilon_{m}\left(\omega \right)\approx 1-\omega_{e}^{2}/\omega^{2}$ with two critical conditions, i.e., $\varepsilon_{m}\left(0\right)=\infty$ and $\varepsilon_{m}\left(\infty \right)=1$, which is valid for plasma and metal. Then, $A_{m}:v$ eventually yields

(8c) $$A_{m}:v=\frac{3}{4}k_{B}T+\frac{3\hbar \omega_{e}}{16\sqrt{2}}.$$

Obviously, $A_{p}:m$ obtained from either Equations (8a) and (8b) is always positive with a typical value ranging in $10^{-19}∼10^{-20}\;J$, demonstrating an ever attractive VDW forces between two nanoparticles of the same material through a melt (taking negative value as attraction and positive one as repulsion for all nanoparticles if without further interpretation).

Unlike in the aqueous solution, electrostatic repulsion in the molten metal has rarely been studied. Although there are works [29,47] assuming negligible electrostatic interaction due to the strong screening effect of background free electrons in the melt, there is reported evidence of charged surface on the oxide nanoparticle in the liquid metal [49,50]. Due to accompanied electron defects (like quasi-free electrons or holes) in the oxide, electron flow occurs across the interface when the oxide nanoparticles are in contact with the liquid metal, resulting in the surface potential $\psi_{i}$ and corresponding profiles at the interface [49,51], as shown in Figure 1c. Combining the above viewpoints from existing studies, the following assumptions are made in the work to model the electrostatic interaction between nanoparticles through melt:

1. Electrons in the molten metal behaves as a free electron gas with a density $n_{e}$, receiving only the contributions from valence electrons of all metallic elements in the melt.
2. Charged surface exists on the oxide nanoparticles in the melt, even though its effect is weak or absent due to the strong screening effect. The value of this surface charge $\psi_{i}$ is assumed to be equal to that obtained from a neutral aqueous dispersion.

Then, the screened-Coulombic potential between two nanoparticles, applied for electrostatic interaction in the electron-screened system (like plasma), is adopted from [66], i.e.

(9) $$U_{E}\left(d_{C}\right)=Z\frac{r_{i}r_{j}}{d_{C}}\exp\left[\frac{d_{C}-\left(r_{i}+r_{j}\right)}{\lambda_{P}}\right],$$

where the interaction coefficient *Z* is analogous to the Hamaker coefficient, reading as

(10) $$Z=4\pi \varepsilon_{0}\psi_{i}\psi_{j},$$

which also presents a positive value for nanoparticles of the same material in the melt, demonstrating an ever repulsive electrostatic interaction. $\lambda_{P}$ is the plasma Debye length, which depends on the free electron density $n_{e}$, i.e.,

$$\lambda_{P}=\sqrt{\frac{\varepsilon_{0}k_{B}T_{M}}{n_{e}e_{0}^{2}}},\;n_{e}=\varrho_{m}N_{A}\frac{\sum_{l}c_{l}z_{l}}{\sum_{l}c_{l}m_{l}},$$

where $z_{l}$, $c_{l}$, and $m_{l}$ are the valence electron number, the atom fraction and the atom mass of the element *l*, respectively. $N_{A}$ is the Avogadro constant, $\varepsilon_{0}$ is the vacuum permittivity, and $e_{0}$ is the elementary charge. Taking the formulation of the interaction potentials in Equations (7) and (9), the VDW and electrostatic force densities are formulated as

(11) $$\begin{array}{cc} f_{A} & =-\frac{32A_{p}:md_{C}{\left(r_{i}r_{j}\right)}^{3}}{3V_{i}{\left[d_{C}^{4}-2d_{C}^{2}\left(r_{i}^{2}+r_{j}^{2}\right)+{\left(r_{i}^{2}-r_{j}^{2}\right)}^{2}\right]}^{2}}\frac{x_{i}-x_{j}}{d_{C}},\\ f_{E} & =Z\frac{r_{i}r_{j}\left(d_{C}+\lambda_{P}\right)}{V_{i}\lambda_{P}d_{C}^{2}}\exp\left[-\frac{d_{C}-\left(r_{i}+r_{j}\right)}{\lambda_{P}}\right]\frac{x_{i}-x_{j}}{d_{C}}. \end{array}$$

According to its physical meaning, $\lambda_{P}$ characterizes the strength of the screening effect due to the electrostatic interaction received by one nanoparticle, which is reflected by the decaying length scale of the $f_{E}$, as shown in Figure 1d. It is calculated that for the alloy such as PM2000, $\lambda_{P}$ is at the scale of $10^{-3}\;\mathrm{nm}$, indicating a very short-range electrostatic repulsion. In addition, $f_{E}$ trends to the ordinary Coulombic potential $f_{E}^{\mathrm{Coul}}$ with a finite value when two nanoparticles are getting in touch, while $f_{A}$ tends to infinity. This demonstrates that the attractive VDW interaction dominates the resultant effect between two nanoparticles, which would destabilize the dispersion system from a perspective of colloidal science [29,48,65] and lead to agglomeration of the nanoparticles. This is against the preference of forming a homogeneous oxide dispersion during the LPBF process. Therefore, the validation of the proposed model relies on further experimental insights into the interactive behavior of the nanoparticle in the molten/liquid metal, which would be covered in our future works.

## 3. Methods

### 3.1. Numerical Scheme and Implementation

**Figure 2:**
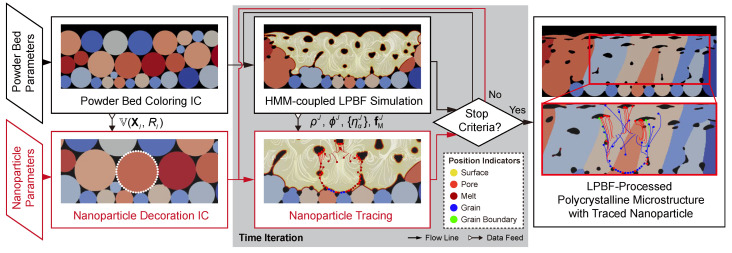
Workflow of the simulation scheme, including a phase-field simulation and a subsequent nanoparticle tracing program.

Assuming negligible counter-effect of the nanoparticles on the melt flow, the simulation is designed in a subsequent scheme: an HMM-coupled phase-field simulation for the LPBF process and a subsequent nanoparticle kinematics simulation with information received from the phase-field simulation, as shown in Figure 2. The information includes the initial condition (IC) of the powder bed as a set $V(X_{I},R_{I})$ with the center $X_{I}$ and the radius $R_{I}$ of the powder *I* to create IC (centers and radii) for the additivated nanoparticles, and nodal values ($\rho^{J},\;\phi^{J},\;\left\{\eta^{J}\right\}$ and $f_{M}^{J}$ of nodal *J*) of every time-step to provide the melt-flow driving force as well as the on-site phase information. Due to the infinitesimal-volume assumption, the drifted nanoparticles are represented and traced by corresponding center position $x_{i}$. The trajectories of the nanoparticles are then calculated numerically by discretizing the kinematic model outlined in Section 2.2 in the backward differences fashion:

(12) $$\begin{array}{cc} v_{i}{|}_{t} & =v_{i}{|}_{t-\delta t}+\left[g+\frac{1}{\varrho_{i}}\left(f_{M}+f_{A}+f_{E}\right)\right]\delta t\\ x_{i}{|}_{t} & =x_{i}{|}_{t-\delta t}+\frac{1}{2}\left[v_{i}{{|}_{t-\delta t}+v_{i}|}_{t}\right]\delta t. \end{array}$$

Note here the time difference δt should be by default no larger than the time interval Δt of the phase-field simulation to ensure the accuracy of the tracing. This tracing program repeats along with the phase-field simulations till reaching the stop criteria, i.e., the end time of simulation or interrupted due to non-converge situation during finite element method calculation. In addition to the trajectories, another important piece of information is the relative position of a nanoparticle in mesoscopic microstructure, for instance, grain interior, grain boundary region, pore, or surface. The position of a nanoparticle can change during its drift and is thus described by a chronological variable, which is termed here as “position indicator”.

The phase-field model is numerically implemented via the finite element method within the program NIsoS developed by authors based on MOOSE framework (Idaho National Laboratory, ID, USA) [67]. Four-node quadrilateral Lagrangian elements are chosen to mesh the geometry. The Cahn-Hilliard equation is solved in a split way [68,69]. A transient solver with preconditioned Jacobian-Free Newton-Krylov (PJFNK) method and second-order backward Euler algorithm has been employed to solve the non-isothermal phase-field problems. Adaptive meshing and time-stepping schemes are used to reduce the computation costs. In addition, to stabilize the calculation of NSCH and NSAC systems, streamline-upwind Petrov-Galerkin and pressure-stabilized Petrov-Galerkin methods are introduced associated with the weak forms of the Navier-Stokes equations [70]. For now, the subsequent nanoparticle tracing program is coded by Python (ver. 3.7.10), which is independent of NIsoS. It is planned to integrate the tracing program as a subroutine of the phase-field simulation program in the future.

### 3.2. Simulation Setup

As a preliminary step, we apply first our nanoparticle tracking scheme for a 2D phase/field simulation of LPBF following our former work [46], which can recapture certain characteristic powder bed features, e.g., particles with multiple sizes and various pores due to the particle packing. The simulation domain has a size of 500×100μm. Particles inside the domain are generated with the random close packing procedure. Due to the uncertainty of the initial grain structure of a single particle, we simply treat each particle as a monocrystal with a unique random orientation following the reported simulation works [46,71]. With the help of the minimum coloring algorithm and grain tracking algorithm [53,72], five $\eta_{\alpha }$ are sufficient to uniquely represent all the particles/grains for these simulations. The zero Neumann boundary condition (BC) for ρ and zero Dirichlet BC for u, representing a close BC for the system, are applied on the boundary Γ=∂Ω of the whole simulation domain Ω,

(13) $${\left.\nabla \rho \right|}_{\Gamma }\;\cdot \;\hat{n}=0,\;{\left.u\right|}_{\Gamma }=0,$$

where $\hat{n}$ is the normal vector of the boundary Γ, and 0 is the null vector. The heat convective BC allows heat dissipation as heat convection with the atmosphere on the top boundary $\Gamma^{\prime }$

(14) $$-{\left.k\nabla T\right|}_{\Gamma^{\prime }}\;\cdot \;\hat{n}=h\left({\left.T\right|}_{\Gamma^{\prime }}-T_{E}\right),$$

where *h* is the convective coefficient and $T_{E}$ is the environment temperature. The Dirichlet BC on temperature with a fixed pre-heating temperature $T_{P}$ is applied on the rest of the boundary $\Gamma^{\prime \prime }$ ($\Gamma =\Gamma^{\prime }\cup \Gamma^{\prime \prime }$) to emulate the contact with a semi-infinite heat reservoir (e.g., the substrate)

(15) $${\left.T\right|}_{\Gamma^{\prime \prime }}=T_{P},$$

which helps to restrain the melt pool size for better demonstration in this work.

In addition, the pinning pressure BC on the top-left corner *C* ($C\in \Gamma^{\prime }$) is applied on the hydrodynamic pressure *p* as

(16) $${\left.p\right|}_{C}=0$$

to avoid the difficulties associated with the non-trivial nullspace of the operator pre-specified in the PJFNK solver as suggested in Ref. [70].

### 3.3. Parameters and Properties

The dimensionless quantities are employed in the nonlinear kinetic system for HMM-coupled phase-field simulations as explained in Section 2.1. Following the conventions of NSCH and NSAC system, they are formulated explicitly as

(17a) Re=ϱv¯l¯ν,Fr=ϱv¯bt¯,Ste=crT¯L,Peρ=v¯ℓl¯γ¯M,PeT=v¯l¯crk,Acϕ=v¯ℓγ¯l¯Lϕ,Acη=v¯ℓγ¯l¯Lη,

where $\bar{v}$, $\bar{l}$ and $\bar{T}$ are the characteristic velocity, length and temperature of the melt flow, while *ℓ* is characteristic width of the diffusive interface corresponding to the given characteristic surface tension $\bar{\gamma }$. ν is dynamic viscosity, *k* is the thermal conductivity, and *M* is the isotropic diffusivity. $L_{\phi }$ and $L_{\eta }$ are the isotropic Allen-Cahn mobility of the solid-liquid interface and grain boundaries. *b* is the magnitude of the resultant body forces acting on the melt flow. For convenience, we use a set of simpler reference quantities to re-define those characteristic quantities by substituting the following relations in Equation (17a):

$$\bar{v}=\frac{\bar{l}}{\bar{t}},\;\bar{T}=T_{M},\;\bar{\gamma }=\sqrt{T_{M}\kappa_{\rho }^{T_{M}}{\underline{C}}_{\mathrm{pt}}^{T_{M}}},\;\ell =\sqrt{\frac{T_{M}\kappa_{\rho }^{T_{M}}}{{\underline{C}}_{\mathrm{pt}}^{T_{M}}}}.$$

Notice that the ${\underline{C}}_{\mathrm{pt}}^{T_{M}}=\kappa_{\rho }^{T_{M}}T_{M}/{\bar{l}}^{2}$ which is the model parameter obtained at the reference temperature $T_{M}$. $\kappa_{\rho }^{T_{M}}$ is the gradient model parameter at a reference temperature $T_{M}$. Then, dimensionless quantities in Equation (17a) can be thereby modified as

(17b) Re=ϱl¯2t¯ν,Fr=ϱl¯bt¯2,Ste=crTML,Peρ=l¯2Mt¯C_ptTM,PeT=l¯2crt¯k,Acϕ=1Lϕt¯C_ptTM,Acη=1Lηt¯C_ptTM.

Notice here that material properties $c_{r}$, *k*, ϱ, ν, should be phase-dependent and thereby formulated in a direct interpolated fashion as

(18) $$\begin{array}{cc} c_{r} & =\Phi_{S}c_{S}^{p}\varrho_{S}+\Phi_{L}c_{L}^{p}\varrho_{L}+\Phi_{\mathrm{at}}c_{\mathrm{at}}^{p}\varrho_{\mathrm{at}},\\ k & =\Phi_{S}k_{S}+\Phi_{L}k_{L}+\Phi_{\mathrm{at}}k_{\mathrm{at}},\;\\ \varrho & =\Phi_{S}\varrho_{S}+\Phi_{L}\varrho_{L}+\Phi_{\mathrm{at}}\varrho_{\mathrm{at}},\;\\ \nu & =\Phi_{S}\nu_{S}+\Phi_{L}\nu_{L}+\Phi_{\mathrm{at}}\nu_{\mathrm{at}}, \end{array}$$

where $c_{(}^{p}\;\cdot \;)$, $k_{(}\;\cdot \;)$, $\varrho_{(}\;\cdot \;)$, and $\nu_{(}\;\cdot \;)$ are respectively the specific enthalpy, thermal conductivity, density, and dynamic viscosity of the corresponding phase. Similarly, the effective value of mobility $L_{\eta }$ and *M* through possible paths are adopted correspondingly from the self-diffusivities $D_{\left(\;\cdot \;\right)}^{\mathrm{eff}}$ and grain boundary mobility $G_{\mathrm{gb}}^{\mathrm{eff}}$ [46,53,73,74], i.e.,

(19) $$\begin{array}{cc} M & =\frac{1}{\partial^{2}f_{\mathrm{loc}}/\partial \rho^{2}{|}_{\rho =1}}\left(\Phi_{\mathrm{ss}}D_{\mathrm{ss}}+\Phi_{\mathrm{at}}D_{\mathrm{at}}+\Phi_{\mathrm{sf}}D_{\mathrm{sf}}+\Phi_{\mathrm{gb}}D_{\mathrm{gb}}\right),\\ L_{\eta } & =\frac{G_{\mathrm{gb}}\gamma_{\mathrm{gb}}}{T\kappa_{\eta }}, \end{array}$$

while the quantity of $L_{\phi }$ is tentatively given as $20/\bar{t}{\underline{C}}_{\mathrm{pt}}^{T_{M}}$, which makes resultant $1/{\mathrm{Ac}}_{\phi }$ sufficiently larger than $1/{\mathrm{Ac}}_{\eta }$ to emulate a relatively faster melting-solidifying process than grain growth [46]. This is due to the lack of a quantitative description of migration mobility of the liquid. The subscript represents the quantities of the corresponding phases, e.g., ‘ss’ as the substance, ‘at’ as the atmosphere/pore, ‘sf’ as the surface, ‘gb’ as the grain boundary, ‘S’ as the solid and ‘L’ as the liquid/melt. Then, the interpolating functions $\Phi_{\mathrm{ss}}$, $\Phi_{\mathrm{at}}$, $\Phi_{\mathrm{gb}}$, $\Phi_{\mathrm{sf}}$, $\Phi_{S}$, and $\Phi_{L}$ can be simply formulated as

(20) $$\begin{array}{cccc} \; & \Phi_{\mathrm{ss}}=\rho^{3}\left(10-15\rho +6\rho^{2}\right), & \; & \Phi_{\mathrm{at}}=1-\rho^{3}\left(10-15\rho +6\rho^{2}\right),\\ \; & \Phi_{\mathrm{sf}}=16\rho^{2}{\left(1-\rho \right)}^{2}, & \; & \Phi_{\mathrm{gb}}=16\sum_{i\neq j}\eta_{i}^{2}\eta_{j}^{2},\\ \; & \Phi_{S}=\phi^{3}\left(10-15\phi +6\phi^{2}\right), & \; & \Phi_{L}=\Phi_{\mathrm{ss}}-\left[\phi^{3}\left(10-15\phi +6\phi^{2}\right)\right]. \end{array}$$

Note that the constraints on the OPs should be also applied on the interpolation functions, i.e., $1=\Phi_{\mathrm{ss}}+\Phi_{\mathrm{at}}$ and $\Phi_{\mathrm{ss}}=\Phi_{L}+\Phi_{S}$.

## 4. Results and Discussion

Simulation results are presented here for a single scan LPBF nanoparticle-decorated PM2000 alloy in an argon atmosphere. The composition of the PM2000 adopted in this work (presented in Table 1 following our former experimental investigations [12,23]) presents a ferritic structure in the high-temperature range (from 1000 K to the melting temperature) without solid-state phase transition, according to the Fe-Cr-Al ternary phase diagram reported in Ref. [75]. Therefore, the material properties for ferritic PM2000 alloy displayed in Table 2 are employed for the simulations. The reference length scale selected for the simulation is $\bar{l}=1\;\mu m$, and the time scale $\bar{t}=1\;\mu s$. The LPBF parameters are selected according to the LPBF experiments in [12]. The characteristic radius of the beam is set as $R_{l}=80\;\mu m$, the penetration depth ζ is defined by the thickness of the powder bed, and the laser power and scanning speed are $P_{l}=160\;W$ and $v_{l}=800\;\mathrm{mm}/s$, respectively. The attenuation coefficient of the laser is β=0.65. The environment temperature $T_{E}$ as well as the pre-heating temperature $T_{P}$ are both set to 353 K.

**Figure 3:**
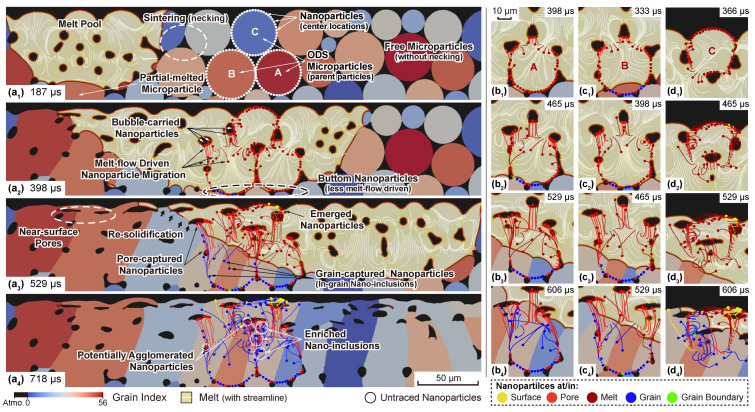
Simulation results of an LPBF-processed ferritic PM2000 powder bed with $P_{l}=160\;W$ and $v_{l}=800\;\mathrm{mm}/s$, in which three particles are marked and decorated with $Y_{2}O_{3}$ nanoparticles. Transient microstructure, nanoparticle tracing, and feature phenomena are presented at (**a${}_{1}$**) 197 s, (**a${}_{2}$**) 398 s, (**a${}_{3}$**) 529 s, (**a${}_{4}$**) 718 s. Trajectories of nanoparticles are respectively depicted for nanoparticles initially located on different parent particle at corresponding timestep, i.e., on parent particle A at (**b${}_{1}$**) 398 s, (**b${}_{2}$**) 465 s, (**b${}_{3}$**) 529 s, (**b${}_{4}$**) 606 s; on parent particle B at (**c${}_{1}$**) 333 s, (**c${}_{2}$**) 398 s, (**c${}_{3}$**) 465 s, (**c${}_{4}$**) 529 s; and on parent particle C at (**d${}_{1}$**) 366 s, (**d${}_{2}$**) 465 s, (**d${}_{3}$**) 529 s, (**d${}_{4}$**) 606 s. Notice that the trace markers at the end of the trajectory only indicate the central location of the nanoparticle at the time rather than the size.

The single scan LPBF of the monomodal $Y_{2}O_{3}$ (15nm in radius) nanoparticle-additivated PM2000 powder is first discussed. Chronological microstructure evolution along with the traced nanoparticles and their trajectory are depicted in Figure 3. To provide a further insight into the steel and nanoparticle evolution during processing, three PM2000 particles from different locations with radii of 17–19 μm are chosen as the “parent particle” and marked as ‘A’, ‘B’, and ‘C’. Fifty equispaced nanoparticles are placed on their surfaces in order to simulate a perfectly homogeneous initial nanoparticle dispersion. Parameters for nanoparticle interactions, including the Hamaker coefficient and the surface potential can be found in Table 3. As the HMM-coupled phase-field simulations [46] show, the microstructure evolution of LPBF-processed PM2000 features multiple phenomena, including melt flow convection as well as accompanied behaviors of pores/bubbles, partially melted particles, re-solidification, and sintering of particles/grains, resulting in columnar grains and trapped irregular-shape pores due to lack of fusion. Those phenomena are also highlighted in Figure 3a${}_{1}$–a${}_{4}$. The focus here is on how the nanoparticles drift and eventually migrate under the influence of those effects. Initially, all the nanoparticles are located onto their corresponding parent particles before the laser interaction and generation of the melt pool (Figure 3a${}_{1}$). Once fully immersed by the melt, the nanoparticle drift is firstly driven by the melt flow, i.e., there is a driving force along the tangent direction of the streamline (Figure 3a${}_{2}$,b${}_{1}$–d${}_{1}$. This effect is illustrated in Figure 1b${}_{1}$–b${}_{4}$). Due to the sufficiently large spacing, other interaction contributions among nanoparticles are negligible. After the initial drift due to the melt front propagation, the evolution of the nanoparticles is associated with different physical phenomena depending on their location and surroundings. Nanoparticles around a pore/bubble, which are identified as the “bubble-carried” ones, would follow the floating, deforming, or even splitting of bubbles (Figure 3a${}_{2}$,b${}_{2}$–d${}_{2}$,b${}_{3}$–d${}_{3}$). It is also possible that the melt flow brings multiple nanoparticles into a narrow region at the time, which is already within the range of nanoparticle interactions (specifically the VDW attraction since the electrostatic repulsion has an even shorter range around $10^{-3}$ nm). In this sense, the trajectory of certain nanoparticles would be redirected abruptly. Meanwhile, nanoparticles near the bottom of the melt pool present very little migration comparing to others. The reason can be the very short immersing time (thus less driven by melt-flow) or the partial melting of the corresponding parent particles. Next, when the laser front scans away, the local temperature drops and, once below $T_{M}$, the re-solidification occurs, forming the tail of the melt pool. Once the re-solidifying front goes through the migrating nanoparticles, they are immediately captured and become either interior or grain boundary (GB) nano-inclusions, while the ones carried by the bubbles stay as they are and become pore-trapped nanoparticles. Nanoparticles can also be found on the surface driven by either melt flow or emerging bubbles (Figure 3a${}_{3}$). Figure 3d${}_{2}$–d${}_{4}$ present an additional case where nanoparticles, initially located on the surface, migrate in accordance with the deformation of the surface morphology. During this process, the nanoparticles with a relatively higher speed (driven by the melt flow) might enter the melt pool and become the in-grain nano-inclusions, while others remain on the surface. Finally, Figure 3a${}_{4}$ presents traced nanoparticles with all sorts of position indicators (denoted by colors) and, notably, the locally enriched nano-inclusions and several ones with overlapped trajectories, implying potential agglomeration effects. One can readily tell from Figure 3a${}_{2}$,a${}_{3}$ that such enrichment majorly can be attributed to the melt-flow driving force, where multiple nanoparticles follow similar trajectories governed by the transient streamlines. However, it is worth noting that the overlap of the trace markers (indicating the center locations of the nanoparticles rather than the sizes) do not sufficiently reflect nanoparticle agglomeration effects, which should be explicitly determined by an adjacency test on the real scale of the nanoparticles’ size. This will be discussed in the following demonstrations.

**Figure 4:**
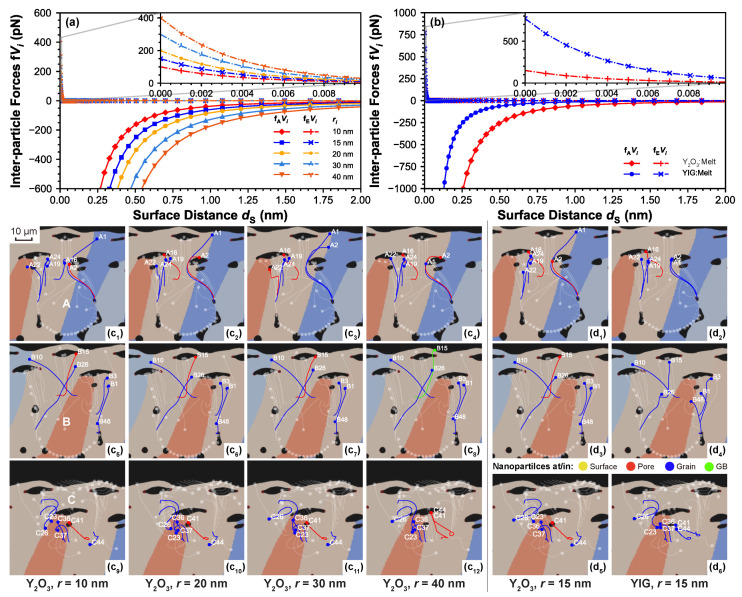
Diagrams of interactive forces between two nanoparticles of the same size vs. surface distance for cases (**a**) varying size (radius) for two $Y_{2}O_{3}$ nanoparticles, and (**b**) comparing nanoparticle compositions of $Y_{2}O_{3}$ and YIG for two nanoparticles with ri = 15 nm. Trajectories and relative positions at the final timestep are respectively for nanoparticles with varying radius and composition on different parent particle, i.e., for $Y_{2}O_{3}$ nanoparticles on parent particle A with varying radius of (**c${}_{1}$**) 10 nm, (**c${}_{2}$**) 20 nm, (**c${}_{3}$**) 30 nm, (**c${}_{4}$**) 40 nm; on parent particle B with radius of (**c${}_{5}$**) 10 nm, (**c${}_{6}$**) 20 nm, (**c${}_{7}$**) 30 nm, (**c${}_{8}$**) 40 nm; on parent particle C with radius of (**c${}_{9}$**) 10 nm, (**c${}_{10}$**) 20 nm, (**c${}_{11}$**) 30 nm, (**c${}_{12}$**) 40 nm; and for nanoparticles with monomodal radius of 15 nm on parent particle A with composition (**d${}_{1}$**) $Y_{2}O_{3}$, (**d${}_{2}$**) YIG; on parent particle B with composition (**d${}_{3}$**) $Y_{2}O_{3}$, (**d${}_{4}$**) YIG; on parent particle C with composition (**d${}_{5}$**) $Y_{2}O_{3}$, (**d${}_{6}$**) YIG. it demonstrates that the pattern of trajectories changes due to varying size/composition. By comparing the selected trajectories (labeled uniformly), an enhanced nanoparticle floating is observed when increasing the size of nanoparticles.

Figure 4a,b presents the interactive forces vs. surface distance between two nanoparticles with different radii and composition, which are $Y_{2}O_{3}$ and YIG, their properties and related coefficients are listed in Table 3. Notice here the Hamaker coefficients in the melt for both $Y_{2}O_{3}$ and YIG are calculated according to Equation (8b) using the coefficients in vacuum for both compositions and the melt, where $A_{m}:v=37\times 10^{-20}\;J$ is calculated by Equation (8c). It can be observed that, for the same surface distance, increasing nanoparticle radius leads to a higher VDW attraction as well as electrostatic repulsion. Comparing nanoparticles of the same size but different composition, YIG nanoparticles present less VDW attraction and more electrostatic repulsion compared to $Y_{2}O_{3}$ ones. Nevertheless, the interaction range of the electrostatic repulsion is limited to the $10^{-3}\;\mathrm{nm}$ scale, and this interaction highly decreases in the nanoscale due to strong screening effects from background free electrons (reflected by very small $\lambda_{P}$), where the VDW attraction still takes the major role. This fact explains the instability of homogeneously dispersed nanoparticles on the 10 nm scale, as explained in Section 2.2. In the case of VDW attraction, it presents a considerable decay when $d_{S}\geq 2\;\mathrm{nm}$, hence its influence over the inter-nanoparticle attractions is low, especially when the nanoparticles are sparsely distributed. Therefore, melt-flow-driven effects are the dominant mechanism, and the effects from nanoparticles’ size (radius) and density would be significant.

In Figure 4c${}_{1}$–c${}_{12}$,d${}_{1}$–c${}_{6}$, nanoparticles with four different radii and two compositions are labeled uniformly with respect to their corresponding parent particles, in which ones with evident changes in their trajectories and position indicators are screened out. Comparing A1, A2 among Figure 4c${}_{1}$–c${}_{3}$; and B10, B15 among Figure 4c${}_{5}$–c${}_{8}$,d${}_{3}$,d${}_{4}$, a higher tendency to float is presented when increasing the size of nanoparticles of identical composition, or using $Y_{2}O_{3}$ than YIG for nanoparticles of identical size. Pair A1–A2 between Figure 4c${}_{3}$,c${}_{4}$ and pair C41–C44 between Figure 4c${}_{9}$–c${}_{12}$, on the other hand, indicate the potential interaction between particles. In particular, the latter pair presents a merged trajectory after one abrupt redirection, implying a potential agglomeration due to a short-range VDW attraction. A similar effect is also spotted for the case with relatively higher density (i.e., YIG) by comparing again the pair A1–A2 between Figure 4d${}_{1}$,d${}_{2}$ and pair C41–C42 between Figure 4d${}_{5}$,d${}_{6}$, demonstrating the potential enhanced interaction in an increased size for nanoparticles of identical composition/using YIG compared to $Y_{2}O_{3}$ for nanoparticles of identical size. Apart from these effects, a more complicated pattern variation in the overall trajectories change shall be discussed with respect to the change in nanoparticle size/composition. Unfortunately, information for current simulations (noting the nanoparticle are sparsely distributed) remains insufficient to deduct such a pattern change, which will be sufficiently covered in our upcoming works.

**Figure 5:**
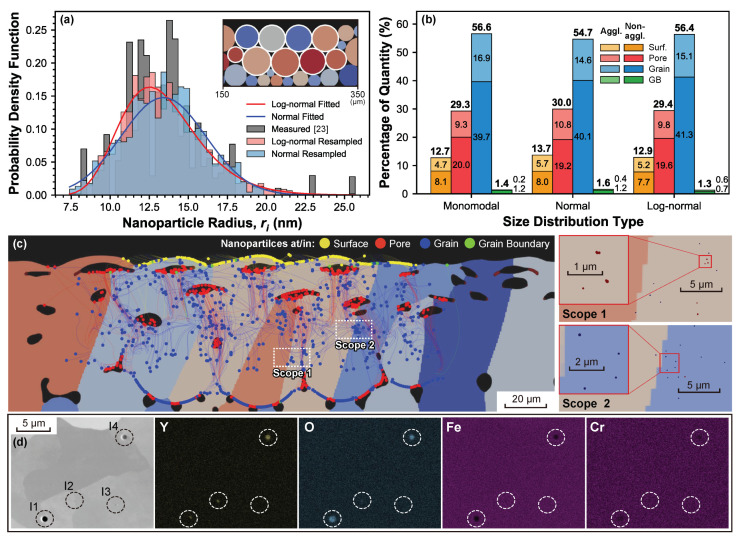
(**a**) Size distributions of nanoparticles. Inset: selected parent particles. (**b**) Statistics of drift destinations of nanoparticles with respect to distinct size distributions, presented as the percentage of quantity to the total amount of decorated nanoparticles, i.e., $N_{p}=1240$, with the ones of agglomerated/non-agglomerated nanoparticles also presented as the component for each destination genre; (**c**) dispersion of traced $Y_{2}O_{3}$ nanoparticles with the log-normal size distribution in the LPBF-processed PM2000 polycrystal matrix (left) and regional magnification of dispersed nanoparticles in selected scopes (Scope 1 and 2) in realistic scale (right). Magnified scopes are employed to distinguish the occurrence of agglomeration from the enrichment, which is inaccessible in the trajectory illustration on the left. (**d**) SEM backscatter electron imaging with the corresponding EDX maps of the elemental Y, O, Fe, and Cr content in four inclusions (I1-I4) for identifying nanoparticle agglomeration. I3 is not agglomerated nanoparticles due to a lack of Y content, while I1 implies agglomerated nanoparticles on the surface of another oxide inclusion. The magnified scope in (**c**) right is also compared to the SEM imaging in (**d**), demonstrating relatively larger nanoparticle agglomeration (I4) spotted in experimental observation, while I2 shows almost a consistent scale to the simulated results.

A statistic investigation of the nanoparticle evolution during processing is conducted to deduct features such as enrichment and agglomeration. The simulations shown in Figure 5 are performed for PM2000 powder with nine selected parent particles decorated with 0.005 wt % $Y_{2}O_{3}$ nanoparticles due to the limited computational efficiency, as presented in the inset of Figure 5a. Notice here that the weight percentage is calculated adapting the 2D scenario, i.e., $\mathrm{wt}\;\%=\frac{N_{p}\varrho_{i}{\bar{r}}_{i}^{2}}{\varrho_{m}R_{I}^{2}}$ with the total amount of nanoparticle $N_{p}$. In this sense, nanoparticles of $N_{p}=1240$ are traced simultaneously. Three types of nanoparticle size distributions are investigated: the normal and the log-normal fittings of the large nanoparticle fraction measured in [23] and a monomodal obtained from the fitted mean radius ${\bar{r}}_{i}=13\;\mathrm{nm}$ of the distribution. To reduce the contamination to the statistics of the drift destinations, alloy powders located lower than the melt pool depth are not decorated with nanoparticles (inset in Figure 5a). Figure 5b presents the statistics of the drift destinations of the $Y_{2}O_{3}$ nanoparticles tested with different size distributions. Employing the adjacency test, in which the center distances ($d_{C}$) between nanoparticles are individually compared with the corresponding sum of mutual radii ($r_{i}+r_{j}$), the agglomerated nanoparticles can be identified. Following this criterion, the nanoparticles are classified as agglomerated/non-agglomerated, showing the corresponding fraction in Figure 5b. The results show that ∼55% of the nanoparticles are captured inside the grains after the LPBF process. Comparing different size distributions, monomodal and log-normal types have almost the same amount of in-grain nanoparticles (nano-inclusions), while the normal type shares the same agglomeration ratio as the log-normal type. However, only ∼2% of the nanoparticles end up in the grain boundaries. Within the nanoparticles in the grain boundaries, the log-normal distribution presents more agglomerated nanoparticles, reaching 46% (0.6% out of in-total 1.3%). In addition, there are nanoparticles that end up in the pores, ∼30%, and the surface, ∼13%. It is worth noting that these statistical results with a high fraction of in-grain nano-inclusions and a very low fraction of GB-captured ones may be attributed to the lack of driving effects from the migration of various interfaces, such as melt-grain interface and grain boundaries, which might force some of the nanoparticles to translate towards the migrating directions, pushing them away from the grain interior and eventually capturing them after the fusion of the powder bed.

In addition to the results provided in Figure 5b, the nanoparticle evolution during processing is graphically displayed in Figure 5c. This methodology is not only useful to predict the nanoparticle agglomeration but can be also extended to study the nanoparticle capture, enrichment, and agglomeration. However, even the evaluation of the agglomeration requires attentions on not only the trajectories overlapping but also the vast differences in spatial scales between the microstructure and the nanoparticles. For instance, Figure 5c presents the trajectories and positions of nanoparticles (log-normal type) dispersed in the LPBF-processed PM2000 polycrystalline matrix, in which two scopes are taken and magnified to visualize the nanoparticle scale. If only the trajectories are evaluated, one might prematurely conclude that Scope 2 has profound agglomerations comparing to Scope 1. However, after a magnified look, agglomerated nanoparticles only appear in Scope 1, while Scope 2 only contains locally enriched isolated nanoparticles.

The issue of identifying the nanoparticle agglomeration/enrichment should be also addressed in the experimental observation, yet the large spatial scale difference makes it difficult to characterize both the steel microstructure and nanoparticle size and dispersion in a single measurement. An example of an experimental SEM-EDX measurement from an LPBF processed sample of PM2000 steel decorated with 0.08 wt % $Y_{2}O_{3}$ is presented in Figure 5d. Four inclusions (labeled as I1-I4) that can be initially thought of as nanoparticle agglomerations during processing can be observed [12]. However, to evaluate whether the inclusions are pores or agglomerated nanoparticles, an elemental mapping with techniques such as EDX is required. The EDX maps show that, while no Y or O content is found in I3, these elements are present in I1, I2, and I4, and so it can be concluded that they are nanoparticle agglomerated structures. Furthermore, there is solely a small fraction of Y content found on the surrounding of I3 while O content is fully presented in the interior, implying agglomerated $Y_{2}O_{3}$ nanoparticles on the surface of another oxide inclusion that might be attributed to impurities. Another interesting point is that the diameter of some nanoparticle agglomerations spotted in Scope 1 of Figure 5c are less than 1 μm, consisting of merely countable nanoparticles, while the ones (esp. I4 in Figure 5d) experimentally observed are larger, and even much larger if compared with the original size of the $Y_{2}O_{3}$ nanoparticles—even though there are some (e.g., I2 in Figure 5d) showing consistency in scale. A possible reason is the relatively low mass fraction of the additivated nanoparticles in the tracing simulation (0.005%) compared to the experimental one (0.08%), which increases the probability of the nanoparticle interactions leading to agglomeration during processing. In future steps, the nanoparticle concentration employed in the simulation will be increased to match the experimental conditions.

## 5. Conclusions

In this work, we proposed a simulation scheme joining the heat-melt-microstructure–coupled phase-field model and the nanoparticle kinematics to trace nanoparticle during the LPBF process of the ODS steels, which is experimentally inaccessible. Simulations on stable LPBF single scan of a ferritic PM2000 nanoparticle-additivated powder bed were conducted for factors such as the nanoparticle composition and size distribution. The following conclusions can be drawn from this combined numerical and experimental study:

1. Simulation results provide the chronological location and located phases of the traced nanoparticle. This helps to depict nanoparticle drift associated with the evolution of local melt flow as well as the morphology, such as migrating nanoparticles driven by melt-flow or carried by floating/deforming bubbles, or stationary ones in the melt pool bottom area. Events such as nanoparticle capture (by grain/pore/grain boundary), enrichment, and potential agglomeration can be also visualized via trajectories and position indicator.
2. The drift and interactions of nanoparticles with different sizes and compositions ($Y_{2}O_{3}$ and YIG) are analyzed. By comparing the trajectories and positions of selected nanoparticles (or nanoparticle pairs) among cases, some preliminary discussions can be conducted regarding the influences of the nanoparticle size and compositions. An enhanced nanoparticle floating is observed when the size is increased for nanoparticles of identical composition or when using $Y_{2}O_{3}$ for nanoparticles of identical size. In addition, an enhanced nanoparticle interaction is observed when increasing the size of nanoparticles of identical composition or using YIG for nanoparticles of identical size. Note that the above conclusions are made under the condition that nanoparticles are sparsely decorated (i.e., nanoparticles are sufficiently spaced). In this scenario, the melt-flow-driven effect is expected to dominate the process.
3. LPBF simulations of a PM2000 powder bed are conducted, in which nine parent particles are additivated with 0.005% $Y_{2}O_{3}$ nanoparticles. Three size distributions are evaluated i.e., monomodal, normal, and log-normal distributions. The results show that ∼55% of the nanoparticles are eventually captured by a grain, while merely ∼2% ones end up in the grain boundaries. Although the differences of nanoparticle location for the different size distributions are small, the monomodal case presents a relatively higher agglomeration ratio in grain-captured nanoparticles (nano-inclusions), while the log-normal type shows a higher agglomeration ratio in GB-captured nanoparticles.
4. By visualizing the traced nanoparticles on a nanometric scale, nanoparticle agglomeration and enrichment spotted in the simulation are distinguished. Comparisons between the simulations and experimental results show promising similarities, proving the potential of the simulation methodology to optimize the LPBF process parameters in order to reduce agglomeration effects and maximize the material reinforcement achieved in ODS steels.

The proposed scheme and models should be further extended in the near future for different aspects, e.g., implementation of nanoparticle tracing code in a computationally-efficient way to enable decoration with a larger mass fraction of the nanoparticles. Further effects such as the driving forces from migrating interfaces, and interactions between nanoparticles and the surface of the parent particles (as ideally the semi-infinity large interfaces) should also be considered.

## Figures and Tables

**Table 1:** Chemical composition of the PM2000 alloy as measured by XRF (only elements present in an amount ≥0.01wt% are shown) [12,23].

	Fe	Cr	Al	Ti	Ni	Si	Cu
wt %	Bal.	20.40	3.94	0.58	0.10	0.03	0.01

**Table 2:** Material properties of the ferritic PM2000 alloy and Ar atmosphere, employed in the simulations.

Properties	Expressions (*T* in K)	Units	References
$T_{M}$	∼1756.15	K	[76]
$\gamma_{\mathrm{sf}}^{\exp}$	$1.63-4.49\times 10^{-3}\left(T-T_{M}\right)$ ${}^{*}$	J/m${}^{2}$	[77,78]
$\gamma_{\mathrm{gb}}^{\exp}$	$0.28-7.74\times 10^{-3}\left(T-T_{M}\right)$ ${}^{*}$	J/m${}^{2}$	[77,78]
$D_{\mathrm{sf}}$	$10\exp(-2.41\times 10^{5}/RT)$ ${}^{†}$	m${}^{2}$/s	[77]
$D_{\mathrm{gb}}$	$1.1\times 10^{-2}\exp\left(-1.74\times 10^{5}/RT\right)$ ${}^{†}$	m${}^{2}$/s	[77]
$D_{\mathrm{ss}}$	$1.8\times 10^{-5}\exp\left(-2.08\times 10^{5}/RT\right)$ ${}^{†}$	m${}^{2}$/s	[77]
$G_{\mathrm{gb}}$	$5.36\exp(-3.54\times 10^{5}/RT)$	m${}^{4}$/(J s)	[79]
$k_{\mathrm{ss}}$	$30.841+0.011(T-T_{M})$	J/(s m K)	[76]
$k_{\mathrm{at}}$	∼0.06	J/(s m K)	[80]
$h_{\mathrm{at}}$	∼100	J/(s m${}^{2}$ K)	
$c_{\mathrm{ss}}^{p}$	$908.596+0.323(T-T_{M})$	J/(kg K)	[76]
$c_{\mathrm{at}}^{p}$	520	J/(kg K)	[81]
L	$2.4\times 10^{9}$ ${}^{†}$	J/m${}^{3}$	[82]
$\varrho_{\mathrm{ss}}$	7180	kg/m${}^{3}$	[76]
$\varrho_{\mathrm{at}}$	1.38	kg/m${}^{3}$	
$\nu_{\mathrm{ss}}$	$∼5.33\times 10^{-3}$ ${}^{‡}$	(J s)/m${}^{3}$	[83]
$\nu_{\mathrm{at}}$	$∼7.53\times 10^{-5}$	(J s)/m${}^{3}$	[84]

* Temperature-dependent data form [78] and scaled based on the value at *T*_M_ from [77]. † Data from ferrite. ‡ Linearly interpolated from temperature-dependent measurements on Fe-Cr melt with 21 at% Cr.

**Table 3:** Material properties and interaction coefficients of the $Y_{2}O_{3}$ and YIG nanoparticles.

	$\varrho_{i}$ (kg/m${}^{3}$)	$A_{p}:v$ ( $10^{-20}\;J$)	$A_{p}:m$ ( $10^{-20}\;J$)	$\psi_{i}$ (mV)	*Z* ( $10^{-14}N$)
$Y_{2}O_{3}$	$5.01\times 10^{3}$	14.0	5.43	26	7.52
YIG	$5.17\times 10^{3}$	24.2	1.33	59	38.73

## Data Availability

The authors declare that the data supporting the findings of this study are available within the paper.

## Abbreviations

The following abbreviations are used in this manuscript:

BC	Boundary condition
DED	Direct energy deposition
EDX	Energy dispersive X-ray spectroscopy
GB	Grain boundary
HMM	Heat-melt-microstructure
IC	Initial condition
LAM	Laser-based additive manufacturing
LPBF	Laser powder bed fusion
NSAC	Navier-Stokes-Allen-Cahn
NSCH	Navier-Stokes-Cahn-Hilliard
ODS	Oxide dispersion strengthened
OP	Order parameter
PJFNK	Preconditioned Jacobian-Free Newton-Krylov
SEM	Scanning electron microscope
VDW	Van der Waals
YIG	Yttrium iron garnet

## Symbols

The following symbols are used in this manuscript:

ρ	Conserved order parameter indicating the substance and
	pores/atmosphere
$\phi_{S}$, $\phi_{L}$, ϕ	Non-conserved order parameters indicating the solid ($\phi_{S}$)
	and liquid ($\phi_{L}$) phase. Due to the substance constraint
	$\phi_{L}=\rho -\phi_{S}$, only one ϕ is sufficient to represent the solid
	substance and (ρ−ϕ) to represent
	liquid substance
$\left\{\eta_{\alpha }\right\}$	Non-conserved order parameters indicating grain
	orientations
u	Velocity field of melt flow
*p*	Hydrodynamic pressure
*T*	Temperature field
$T_{M}$, $T_{E}$, $T_{P}$	Melting temperature, environmental temperature, and
	pre-heating temperature, respectively
$f_{\mathrm{loc}},f_{\mathrm{grad}}$, $f_{ht}$	Local, gradient and thermal terms of Helmholtz free energy
	density
$\underline{A},\underline{B},\underline{G}$	Model parameters with no dimension
${\underline{C}}_{\mathrm{pt}},{\underline{D}}_{\mathrm{pt}},{\underline{H}}_{\mathrm{pt}}$	Model parameters with dimension of free energy density
${\underline{C}}_{\mathrm{cf}},{\underline{D}}_{\mathrm{cf}},{\underline{H}}_{\mathrm{cf}}$	Model parameters with dimension of entropy density
$\kappa_{\rho }$, $\kappa_{\phi }$, $\kappa_{\eta }$	Gradient constants of corresponding order parameters
$\Phi_{\mathrm{ss}},\Phi_{\mathrm{at}},\Phi_{\mathrm{sf}},\Phi_{\mathrm{gb}}$, $\Phi_{S},\Phi_{L}$	Interpolation functions with subscript ‘ss’ standing for the
	substance, ‘at’ for the atmosphere/pore, ‘sf’ for the surface,
	‘gb’ for the grain boundary, ‘S’ for the solid and ‘L’ for the
	liquid/melt
$P_{l}$, $v_{l}$, $v_{l}$	Laser power, scan velocity and speed, respectively. $v_{l}=\left|v_{l}\right|$
$R_{l}$	Nominal laser radius
$q_{v}$	Internal heat source for modeling the laser-induced thermal
	effect
β	Attenuation coefficient of the powder bed
ζ	Characteristic penetration depth of the powder bed
*h*	Heat convective coefficient
$k_{S},k_{L},k_{\mathrm{at}}$	Heat conductivity coefficients with subscripts indicating the
	corresponding phases
$\nu_{S},\nu_{L},\nu_{\mathrm{at}}$	Dynamic viscosity coefficients with subscripts indicating
	the corresponding phases
$\varrho_{S},\varrho_{L},\varrho_{\mathrm{at}}$	Densities with subscripts indicating the corresponding
	phases
$c_{S}^{p},c_{L}^{p},c_{\mathrm{at}}^{p}$	Specific enthalphies with subscripts indicating the
	corresponding phases
$c_{r}$	Relative volumetric specific heat
L	Latent heat during melting/solidification
$G_{\mathrm{gb}}^{\mathrm{eff}}$	Grain boundary mobility
$D_{\mathrm{ss}},D_{\mathrm{at}}$, $D_{\mathrm{sf}},D_{\mathrm{gb}}$	Self-diffusivity coefficients with subscripts indicating the
	corresponding phases
*M*	Isotropic Cahn–Hilliard mobility
$L_{\phi }$, $L_{\eta }$	Isotropic Allen–Cahn mobilities
*b*	Magnitude of the resultant body forces acting on the melt
	flow
Re,Ste,PeT, ${\mathrm{Pe}}_{\rho },{\mathrm{Ac}}_{\phi },{\mathrm{Ac}}_{\eta }$	Reynold number, Froude number, Stefan number, Péclet
	numbers for thermal and mass transfer, Allen–Cahn
	numbers for melting-solidification and grain growth,
	respectively
$V_{i},\varrho_{i},v_{i}$	Volume, density and translation velocity of the *i*-th
	dispersed rigid nanoparticle in the melt respectively
$f_{M},f_{A},f_{E}$	Driving force densities due to the melt flow, the VDW and
	the electrostatic interaction, respectively
σ	Cauchy stress tensor of the melt
$d_{C},d_{S}$	Inter-particle center distance and surface distance,
	respectively
$U_{A}$, $U_{E}$	Inter-particle potential of VDW and the electrostatic
	interactions, respectively
$A_{p}:v,A_{m}:v,A_{p}:m$	Hamaker coefficients with subscript ‘p:v’ standing for
	nanoparticle in the vacuum, ‘m:v’ for medium/melt in the
	vacuum, and ‘p:m’ for nanoparticle in the medium/melt
*Z*	Screened-Coulombic interaction coefficient
$\psi_{i}$	Nanoparticle surface potential
$\varepsilon_{p}$, $\varepsilon_{m},\varepsilon_{0}$	Permittivities of the nanoparticle, medium and vacuum,
	respectively
$N_{p}$	Total amount of the nanoparticle,
$n_{p}$, $n_{m}$	Refractive indices of the nanoparticle and the medium,
	respectively
$\lambda_{P}$	Plasma Debye length
$n_{e}$, $\omega_{e}$	Free electron density and electronic absorption frequency
	of the melt
$z_{l}$, $c_{l}$, $m_{l}$	Valence electron number, atom fraction and the atom mass
	of the *l*-th element in the alloy, respectively
$k_{B},\hbar ,e_{0},N_{A}$	Boltzmann constant, reduced Planck constant, elementary
	charge, and Avogadro constant, respectively
$V(X_{I},R_{I})$	Powder bed as a set, containing the center coordinates ($X_{I}$)
	and the radius ($R_{I}$) of *I*-th powder
∇, $\nabla_{d}$	Gradient operators. Subscript ‘*d*’ represents the gradient
	along the inter-particle direction

## References

[1] Martin J.H., Yahata B.D., Hundley J.M., Mayer J.A., Schaedler T.A., Pollock T.M. (2017). 3D printing of high-strength aluminium alloys. Nature.

[2] Kürnsteiner P., Wilms M.B., Weisheit A., Gault B., Jägle E.A., Raabe D. (2020). High-strength Damascus steel by additive manufacturing. Nature.

[3] Gu D., Shi X., Poprawe R., Bourell D.L., Setchi R., Zhu J. (2021). Material-structure-performance integrated laser-metal additive manufacturing. Science.

[4] Orowan E. (1948). Discussion on internal stresses. Symposium on Internal Stresses in Metals and Alloys.

[5] Wharry J.P., Swenson M.J., Yano K.H. (2017). A review of the irradiation evolution of dispersed oxide nanoparticles in the bcc Fe-Cr system: Current understanding and future directions. J. Nucl. Mater..

[6] Jiang Y., Smith J.R., Odette G.R. (2010). Prediction of structural, electronic and elastic properties of Y2Ti2O7 and Y2TiO5. Acta Mater..

[7] Suresh K., Nagini M., Vijay R., Ramakrishna M., Gundakaram R.C., Reddy A., Sundararajan G. (2016). Microstructural studies of oxide dispersion strengthened austenitic steels. Mater. Des..

[8] Schneibel J., Heilmaier M., Blum W., Hasemann G., Shanmugasundaram T. (2011). Temperature dependence of the strength of fine-and ultrafine-grained materials. Acta Mater..

[9] Unocic K.A., Pint B.A., Hoelzer D.T. (2016). Advanced TEM characterization of oxide nanoparticles in ODS Fe–12Cr–5Al alloys. J. Mater. Sci..

[10] Karak S., Chudoba T., Witczak Z., Lojkowski W., Manna I. (2011). Development of ultra high strength nano-Y2O3 dispersed ferritic steel by mechanical alloying and hot isostatic pressing. Mater. Sci. Eng. A.

[11] Chang H.J., Cho H.Y., Kim J.H. (2015). Stability of Y–Ti–O nanoparticles during laser melting of advanced oxide dispersion-strengthened steel powder. J. Alloy. Compd..

[12] Doñate-Buendia C., Kürnsteiner P., Stern F., Wilms M.B., Streubel R., Kusoglu I.M., Tenkamp J., Bruder E., Pirch N., Barcikowski S. (2021). Microstructure formation and mechanical properties of ODS steels built by laser additive manufacturing of nanoparticle coated iron-chromium powders. Acta Mater..

[13] Boegelein T., Louvis E., Dawson K., Tatlock G.J., Jones A.R. (2016). Characterisation of a complex thin walled structure fabricated by selective laser melting using a ferritic oxide dispersion strengthened steel. Mater. Charact..

[14] Vasquez E., Giroux P.F., Lomello F., Nussbaum M., Maskrot H., Schuster F., Castany P. (2020). Effect of powder characteristics on production of oxide dispersion strengthened Fe14Cr steel by laser powder bed fusion. Powder Technol..

[15] Vasquez E., Giroux P.F., Lomello F., Chniouel A., Maskrot H., Schuster F., Castany P. (2019). Elaboration of oxide dispersion strengthened Fe-14Cr stainless steel by selective laser melting. J. Mater. Process. Technol..

[16] Ghayoor M., Lee K., He Y., Chang C.h., Paul B.K., Pasebani S. (2020). Selective laser melting of austenitic oxide dispersion strengthened steel: Processing, microstructural evolution and strengthening mechanisms. Mater. Sci. Eng. A.

[17] AlMangour B., Grzesiak D., Yang J.M. (2016). Rapid fabrication of bulk-form TiB2/316L stainless steel nanocomposites with novel reinforcement architecture and improved performance by selective laser melting. J. Alloy. Compd..

[18] Brocq M., Radiguet B., Poissonnet S., Cuvilly F., Pareige P., Legendre F. (2011). Nanoscale characterization and formation mechanism of nanoclusters in an ODS steel elaborated by reactive-inspired ball-milling and annealing. J. Nucl. Mater..

[19] Cayron C., Rath E., Chu I., Launois S. (2004). Microstructural evolution of *Y*_2_*O*_3_ and *M**g**A**l*_2_*O*_4_ ODS EUROFER steels during their elaboration by mechanical milling and hot isostatic pressing. J. Nucl. Mater..

[20] Bergner F., Hilger I., Virta J., Lagerbom J., Gerbeth G., Connolly S., Hong Z., Grant P.S., Weissgärber T. (2016). Alternative Fabrication Routes toward Oxide-Dispersion-Strengthened Steels and Model Alloys. Metall. Mater. Trans. A.

[21] Smith T.M., Thompson A.C., Gabb T.P., Bowman C.L., Kantzos C.A. (2020). Efficient production of a high-performance dispersion strengthened, multi-principal element alloy. Sci. Rep..

[22] Moghadasi M.A., Nili-Ahmadabadi M., Forghani F., Kim H.S. (2016). Development of an oxide-dispersion-strengthened steel by introducing oxygen carrier compound into the melt aided by a general thermodynamic model. Sci. Rep..

[23] Doñate-Buendía C., Frömel F., Wilms M.B., Streubel R., Tenkamp J., Hupfeld T., Nachev M., Gökce E., Weisheit A., Barcikowski S. (2018). Oxide dispersion-strengthened alloys generated by laser metal deposition of laser-generated nanoparticle-metal powder composites. Mater. Des..

[24] Streubel R., Wilms M.B., Doñate-Buendía C., Weisheit A., Barcikowski S., Schleifenbaum J.H., Gökce B. (2018). Depositing laser-generated nanoparticles on powders for additive manufacturing of oxide dispersed strengthened alloy parts via laser metal deposition. Jpn. J. Appl. Phys..

[25] Springer H., Baron C., Szczepaniak A., Jägle E.A., Wilms M.B., Weisheit A., Raabe D. (2016). Efficient additive manufacturing production of oxide- and nitride-dispersion-strengthened materials through atmospheric reactions in liquid metal deposition. Mater. Des..

[26] Williams C.A., Unifantowicz P., Baluc N., Smith G.D., Marquis E.A. (2013). The formation and evolution of oxide particles in oxide-dispersion- strengthened ferritic steels during processing. Acta Mater..

[27] Li S., Xiao H., Liu K., Xiao W., Li Y., Han X., Mazumder J., Song L. (2017). Melt-pool motion, temperature variation and dendritic morphology of Inconel 718 during pulsed- and continuous-wave laser additive manufacturing: A comparative study. Mater. Des..

[28] AlMangour B., Baek M.S., Grzesiak D., Lee K.A. (2018). Strengthening of stainless steel by titanium carbide addition and grain refinement during selective laser melting. Mater. Sci. Eng. A.

[29] Xu J.Q., Chen L.Y., Choi H., Li X.C. (2012). Theoretical study and pathways for nanoparticle capture during solidification of metal melt. J. Phys. Condens. Matter.

[30] Heiden M.J., Deibler L.A., Rodelas J.M., Koepke J.R., Tung D.J., Saiz D.J., Jared B.H. (2019). Evolution of 316L stainless steel feedstock due to laser powder bed fusion process. Addit. Manuf..

[31] King W.E., Anderson A.T., Ferencz R.M., Hodge N.E., Kamath C., Khairallah S.A., Rubenchik A.M. (2015). Laser powder bed fusion additive manufacturing of metals; physics, computational, and materials challenges. Appl. Phys. Rev..

[32] Zhang D., Qiu D., Gibson M.A., Zheng Y., Fraser H.L., StJohn D.H., Easton M.A. (2019). Additive manufacturing of ultrafine-grained high-strength titanium alloys. Nature.

[33] Khairallah S.A., Anderson A. (2014). Mesoscopic simulation model of selective laser melting of stainless steel powder. J. Mater. Process. Technol..

[34] McCallen C. (2012). Technical Report LLNL-ABS-565212.

[35] Shi R., Khairallah S.A., Roehling T.T., Heo T.W., McKeown J.T., Matthews M.J. (2020). Microstructural control in metal laser powder bed fusion additive manufacturing using laser beam shaping strategy. Acta Mater..

[36] Khairallah S.A., Anderson A.T., Rubenchik A., King W.E. (2016). Laser powder-bed fusion additive manufacturing: Physics of complex melt flow and formation mechanisms of pores, spatter, and denudation zones. Acta Mater..

[37] Körner C., Attar E., Heinl P. (2011). Mesoscopic simulation of selective beam melting processes. J. Mater. Process. Technol..

[38] Attar E., Körner C. (2009). Lattice Boltzmann method for dynamic wetting problems. J. Colloid Interface Sci..

[39] Attar E., Körner C. (2011). Lattice Boltzmann model for thermal free surface flows with liquid–solid phase transition. Int. J. Heat Fluid Flow.

[40] Rai A., Markl M., Körner C. (2016). A coupled Cellular Automaton–Lattice Boltzmann model for grain structure simulation during additive manufacturing. Comput. Mater. Sci..

[41] Rai A., Helmer H., Körner C. (2017). Simulation of grain structure evolution during powder bed based additive manufacturing. Addit. Manuf..

[42] Lian Y., Lin S., Yan W., Liu W.K., Wagner G.J. (2018). A parallelized three-dimensional cellular automaton model for grain growth during additive manufacturing. Comput. Mech..

[43] Rolchigo L. (2019). Application of alloy solidification theory to cellular automata modeling of near–rapid constrained solidification. Comput. Mater. Sci..

[44] Zhang Y., Zhang J. (2019). Modeling of solidification microstructure evolution in laser powder bed fusion fabricated 316L stainless steel using combined computational fluid dynamics and cellular automata. Addit. Manuf..

[45] Yang M., Wang L., Yan W. (2021). Phase-field modeling of grain evolutions in additive manufacturing from nucleation, growth, to coarsening. Npj Comput. Mater..

[46] Yang Y., Kühn P., Yi M., Egger H., Xu B.X. (2020). Non-Isothermal Phase-Field Modeling of Heat–Melt–Microstructure-Coupled Processes during Powder Bed Fusion. JOM.

[47] Xu J. (2015). Achieving Uniform Nanoparticle Dispersion in Metal Matrix Nanocomposites. Doctor of Philosophy in Materials Science and Engineering.

[48] Kamysbayev V., James N.M., Filatov A.S., Srivastava V., Anasori B., Jaeger H.M., Gogotsi Y., Talapin D.V. (2019). Colloidal Gelation in Liquid Metals Enables Functional Nanocomposites of 2D Metal Carbides (MXenes) and Lightweight Metals. ACS Nano.

[49] Paik Y.H., Yoon W.J., Shin H.C. (2004). Static electrification of solid oxide in liquid metal and electrical double layer at the interface. J. Colloid Interface Sci..

[50] Paik Y.H., Pan J.H., Yoon W.J. (2001). Charging phenomena at the metal oxide-liquid metal interfaces and determination of excess electron density of metal oxide-mercury systems by the induced emf method. J. Colloid Interface Sci..

[51] Zeng M., Cao H., Zhang Q., Gao X., Fu L. (2018). Self-Assembly of Metal Oxide Nanoparticles in Liquid Metal toward Nucleation Control for Graphene Single-Crystal Arrays. Chem.

[52] Yang Y., Oyedeji T.D., Kühn P., Xu B.X. (2020). Investigation on Temperature-Gradient-Driven Effects in Unconventional Sintering *Via* Non-Isothermal Phase-Field Simulation. Scr. Mater..

[53] Yang Y., Ragnvaldsen O., Bai Y., Yi M., Xu B.X. (2019). 3D Non-Isothermal Phase-Field Simulation of Microstructure Evolution during Selective Laser Sintering. Npj Comput. Mater..

[54] (2005). Lasers and laser-related equipment–Test methods for laser beam widths, divergence angles and beam propagation ratios–Part 1: Stigmatic and simple astigmatic beams. Standard ISO 11146-1: 2005 (E).

[55] Shukla S., Bhattacharjee S., Weber A.Z., Secanell M. (2017). Experimental and Theoretical Analysis of Ink Dispersion Stability for Polymer Electrolyte Fuel Cell Applications. J. Electrochem. Soc..

[56] Bergström L., Meurk A., Arwin H., Rowcliffe D.J. (1996). Estimation of Hamaker constants of ceramic materials from optical data using Lifshitz theory. J. Am. Ceram. Soc..

[57] Jiang L., Rahnama M., Zhang B., Zhu X., Sui P.C., Ye D.D., Djilali N. (2020). Predicting the interaction between nanoparticles in shear flow using lattice Boltzmann method and Derjaguin-Landau-Verwey-Overbeek (DLVO) theory. Phys. Fluids.

[58] Ladd A., Verberg R. (2001). Lattice-Boltzmann simulations of particle-fluid suspensions. J. Stat. Phys..

[59] Ouchene R. (2020). Numerical simulation and modeling of the hydrodynamic forces and torque acting on individual oblate spheroids. Phys. Fluids.

[60] Glowinski R., Pan T.W., Hesla T.I., Joseph D.D. (1999). A distributed Lagrange multiplier/fictitious domain method for particulate flows. Int. J. Multiph. Flow.

[61] Glowinski R., Pan T.W., Hesla T.I., Joseph D.D., Périaux J. (2001). A Fictitious Domain Approach to the Direct Numerical Simulation of Incompressible Viscous Flow past Moving Rigid Bodies: Application to Particulate Flow. J. Comput. Phys..

[62] Hamaker H.C. (1937). The London-van der Waals attraction between spherical particles. Physica.

[63] Lifshitz E.M., Hamermesh M. (1992). The theory of molecular attractive forces between solids. Perspectives in Theoretical Physics.

[64] Dzyaloshinskii I.E., Lifshitz E.M., Pitaevskii L.P. (1961). The general theory of van der Waals forces. Adv. Phys..

[65] Israelachvili J.N. (2011). Intermolecular and Surface Forces.

[66] Ohshima H. (1994). Electrostatic interaction between two spherical colloidal particles. Adv. Colloid Interface Sci..

[67] Tonks M.R., Gaston D., Millett P.C., Andrs D., Talbot P. (2012). An object-oriented finite element framework for multiphysics phase field simulations. Comput. Mater. Sci..

[68] Elliott C.M., French D.A., Milner F. (1989). A second order splitting method for the Cahn-Hilliard equation. Numer. Math..

[69] Balay S., Gropp W.D., McInnes L.C., Smith B.F. (1997). Efficient management of parallelism in object-oriented numerical software libraries. Modern Software Tools for Scientific Computing.

[70] Peterson J.W., Lindsay A.D., Kong F. (2018). Overview of the incompressible Navier–Stokes simulation capabilities in the MOOSE framework. Adv. Eng. Softw..

[71] Lu L.X., Sridhar N., Zhang Y.W. (2018). Phase field simulation of powder bed-based additive manufacturing. Acta Mater..

[72] Permann C.J., Tonks M.R., Fromm B., Gaston D.R. (2016). Order parameter re-mapping algorithm for 3D phase field model of grain growth using FEM. Comput. Mater. Sci..

[73] Moelans N., Blanpain B., Wollants P. (2008). Quantitative analysis of grain boundary properties in a generalized phase field model for grain growth in anisotropic systems. Phys. Rev. B.

[74] Chockalingam K., Kouznetsova V.G., van der Sluis O., Geers M.G. (2016). 2D Phase field modeling of sintering of silver nanoparticles. Comput. Methods Appl. Mech. Eng..

[75] Chang K., Meng F., Ge F., Zhao G., Du S., Huang F. (2019). Theory-guided bottom-up design of the FeCrAl alloys as accident tolerant fuel cladding materials. J. Nucl. Mater..

[76] MatWeb (2021). Schwarzkopf Plansee PM 2000 Grain Class 4 ODS Iron Alloy. www.matweb.com/search/datasheetprint.aspx?matguid=96b81a5874794095964dae30a476b069.

[77] Hill A., Wallach E.R. (1989). Modelling solid-state diffusion bonding. Acta Metall..

[78] Price A., Holl H., Greenough A. (1964). The surface energy and self diffusion coefficient of solid iron above 1350 C. Acta Metall..

[79] Capdevila C., Chen Y.L., Jones A.R., Bhadeshia H.K. (2003). Grain boundary mobility in Fe-base oxide dispersion strengthened PM2000 alloy. ISIJ Int..

[80] Hoshino T., Mito K., Nagashima A., Miyata M. (1986). Determination of the thermal conductivity of argon and nitrogen over a wide temperature range through data evaluation and shock-tube experiments. Int. J. Thermophys..

[81] Chase M.W. (1998). NIST-JANAF thermochemical tables. J. Phys. Chem. Ref. Data, Monogr..

[82] Liu F., Zhang Q., Zhou W., Zhao J., Chen J. (2012). Micro scale 3D FEM simulation on thermal evolution within the porous structure in selective laser sintering. J. Mater. Process. Technol..

[83] Kamaeva L.V., Sterkhova I.V., Lad’yanov V.I. (2012). Viscosity and supercooling of Fe-Cr (≤40 at % Cr) melts. Inorg. Mater..

[84] Dawe R.A., Smith E.B. (1970). Viscosities of the Inert Gases at High Temperatures. J. Chem. Phys..

